# Activin A Modulates CRIPTO-1/HNF4*α*^+^ Cells to Guide Cardiac Differentiation from Human Embryonic Stem Cells

**DOI:** 10.1155/2017/4651238

**Published:** 2017-01-09

**Authors:** Robin Duelen, Guillaume Gilbert, Abdulsamie Patel, Nathalie de Schaetzen, Liesbeth De Waele, Llewelyn Roderick, Karin R. Sipido, Catherine M. Verfaillie, Gunnar M. Buyse, Lieven Thorrez, Maurilio Sampaolesi

**Affiliations:** ^1^Translational Cardiomyology Laboratory, Stem Cell Biology and Embryology Unit, Department of Development and Regeneration, KU Leuven, 3000 Leuven, Belgium; ^2^Experimental Cardiology, Department of Cardiovascular Sciences, KU Leuven, 3000 Leuven, Belgium; ^3^Stem Cell Institute Leuven and Stem Cell Biology and Embryology Unit, Department of Development and Regeneration, KU Leuven, 3000 Leuven, Belgium; ^4^Child Neurology, University Hospitals Leuven, Department of Development and Regeneration, KU Leuven, 3000 Leuven, Belgium; ^5^Tissue Engineering Laboratory, Department of Development and Regeneration, KU Leuven, Campus Kulak Kortrijk, 8500 Kortrijk, Belgium; ^6^Division of Human Anatomy, Department of Public Health, Experimental and Forensic Medicine, University of Pavia, 27100 Pavia, Italy

## Abstract

The use of human pluripotent stem cells in basic and translational cardiac research requires efficient differentiation protocols towards cardiomyocytes. In vitro differentiation yields heterogeneous populations of ventricular-, atrial-, and nodal-like cells hindering their potential applications in regenerative therapies. We described the effect of the growth factor Activin A during early human embryonic stem cell fate determination in cardiac differentiation. Addition of high levels of Activin A during embryoid body cardiac differentiation augmented the generation of endoderm derivatives, which in turn promoted cardiomyocyte differentiation. Moreover, a dose-dependent increase in the coreceptor expression of the TGF-*β* superfamily member* CRIPTO-1* was observed in response to Activin A. We hypothesized that interactions between cells derived from meso- and endodermal lineages in embryoid bodies contributed to improved cell maturation in early stages of cardiac differentiation, improving the beating frequency and the percentage of contracting embryoid bodies. Activin A did not seem to affect the properties of cardiomyocytes at later stages of differentiation, measuring action potentials, and intracellular Ca^2+^ dynamics. These findings are relevant for improving our understanding on human heart development, and the proposed protocol could be further explored to obtain cardiomyocytes with functional phenotypes, similar to those observed in adult cardiac myocytes.

## 1. Introduction

The generation of functional cardiomyocytes (CMs) differentiated from pluripotent stem cell (PSC) lines offers an extraordinary platform to develop novel cell-based therapies, to establish predictive drug toxicology tests, to model human diseases in vitro, and to study human embryonic development [1]. Strategies to efficiently direct differentiation of human embryonic stem cell (ESC) and induced pluripotent stem cell (iPSC) lines towards cardiovascular lineages are of particular interest due to the high morbidity and mortality of cardiovascular diseases in the Western world. So far, the most successful in vitro differentiation approaches are those that recapitulate the regulatory pathways of embryonic cardiac development (reviewed in [2, 3]).

PSC differentiation to CMs has made considerable progress in the past decade. One of the first directed differentiation protocols described involves the coculture of human ESCs with mouse visceral endoderm-like cells (END-2) [4]. Currently, two basic methods for cardiac differentiation of human PSC lines are in use: differentiation of cultured human PSCs as a monolayer and as embryoid bodies (EBs) (reviewed in [2, 3]).

Studies, using different model organisms, have demonstrated that the morphogenic Activin A (ActA)/NODAL, bone morphogenetic protein (BMP), and Wnt signaling pathways played pivotal roles in the establishment of a cardiovascular cell fate [5–16]. Recently published reports have shown that BMP4 and basic fibroblast growth factor (bFGF) signaling modulated ActA-induced mesendoderm differentiation in mouse [17–19] and human ESC cultures [20]. Moreover, the combinatorial effects of BMP4 and ActA induced cardiovascular development in serum-free human ESCs [21, 22]. Kattman et al. have reported that individual mouse and human PSC lines required optimization for the proper balance of the BMP4 and ActA signaling cascade to achieve efficient cardiac differentiation [23]. However, these studies did not define a stage-specific role for these morphogens nor the influence of different levels of signaling on the differentiation.

BMPs and ActA are members of the transforming growth factor beta (TGF-*β*) superfamily and control a wide range of fundamental cellular processes, including tissue patterning during embryogenesis and cell proliferation and differentiation. These TGF-*β* ligands exert their biological effects by binding and assembling two types of transmembrane receptors (type I and type II) with intrinsic serine/threonine kinase activities [24, 25]. ActA binds to type II receptor, ACVR2A or ACVR2B, leading to oligomerization, which recruits and phosphorylates the activin type I receptor-like kinase 4 (ALK4, or also known as ACVR1B) (reviewed in [26]). ActA and NODAL utilize the same signaling receptors, although their mechanism of ligand-mediated interaction with their receptor is different. NODAL lacks intrinsic affinity for ACVR2A/2B and ALK4 and requires CRIPTO-1, also known as teratocarcinoma-derived growth factor-1 (TDGF1), which belongs to the epidermal growth factor-Cripto-FRL1-Cryptic (EGF-CFC) family, and it has a pivotal role during embryogenesis and tumorigenesis [27]. Studies have shown that NODAL assembled type II and type I receptors only when CRIPTO-1 was present [28, 29]. During mouse embryonic development, Cripto-1 was expressed in the inner cell mass of blastocysts at day 4 and in the primitive streak at day 6.5 [30]. Xu et al. have demonstrated that mouse ESCs lacking Cripto-1 expression lost the ability to form beating CMs in vitro [31]. More interestingly, mouse Cripto-1 deficient embryos died at around day 6.5 due to mesoderm formation defects [32]. Minchiotti et al. have documented that Cripto-1 signaling was crucial for priming differentiation of mouse ESCs into functional CMs [33, 34]. Recently, Fiorenzano et al. provided evidence that CRIPTO-1 was a major determinant of mouse epiblast stem cell (EpiSC) and human ESC pluripotency, suggesting an earlier function of* CRIPTO-1* than previously recognized in the very first lineage decision made by the early embryo [35].

In this study, we investigated the role of the ActA signaling pathway during CM differentiation from human ESCs. The expression of the mesoderm marker* BRACH* and the endoderm-associated markers* SOX17* and* HNF4α* confirmed ActA addition as a robust inducer of mesendoderm formation. We showed that high doses of ActA (50 and 100 ng/mL), added during the initial stage of cardiac differentiation, improved cell maturation in the early stages of differentiation. Specifically, ActA increased the percentage of contracting EBs and the beating frequency of these EBs. CRIPTO-1 acting in the early time window of ESC differentiation might be responsible for priming the CM cell fate. Finally, we demonstrated that early commitment by ActA appeared to improve cardiac cell type specification and that ActA-mediated differentiation of human ESC-derived CMs (ESC-CMs) allowed the production of CMs with atrial- and ventricular-like action potentials (APs) and Ca^2+^ dynamics supporting advanced maturation of the cells. In conclusion, human ESC-derived EBs treated with high ActA concentrations during the first two days of cardiac differentiation responded by increasing numbers of definitive endodermal cells, which in turn promoted CM differentiation and early cell maturation in the early stages of differentiation.

## 2. Material and Methods

### 2.1. Human Embryonic Stem Cell Culture

Human ESC lines, H1 (WA01) and H9 (WA09, both from WiCell Research Institute), were used between passages 43 and 63 and had a normal karyotype, checked by WiCell Cytogenetic Services. To maintain ESCs in culture, human ESCs were cultured on a feeder layer of irradiated mouse embryonic fibroblasts (MEFs, GlobalStem) under hypoxic conditions. Undifferentiated cells on MEFs were cultured in normal maintenance medium for human ESC lines: knockout DMEM/F12 and 20% knockout serum replacement (KSR) supplemented with 2 mM L-glutamine, 1% nonessential amino acids (NEAA), 0.1 mM *β*-mercaptoethanol, 0.5% Pen/Strep (all from Thermo Fisher Scientific), and 10 ng/mL bFGF (PeproTech). Prior to differentiation, the human ESCs were feeder depleted by culturing on a thin growth factor reduced (GFR) Matrigel layer (BD Biosciences) for at least three passages. Feeder-free cultures were in complete serum-free mTeSR medium (STEMCELL Technologies), consisting of basal medium and 5x supplement with recombinant human bFGF and TGF-*β* and maintained under normoxia. NKX2.5^eGFP/w^ human ESC line was kindly provided by Elliott et al. [36].

### 2.2. Human Embryonic Stem Cell Cardiac Differentiation

**Figure 1:**
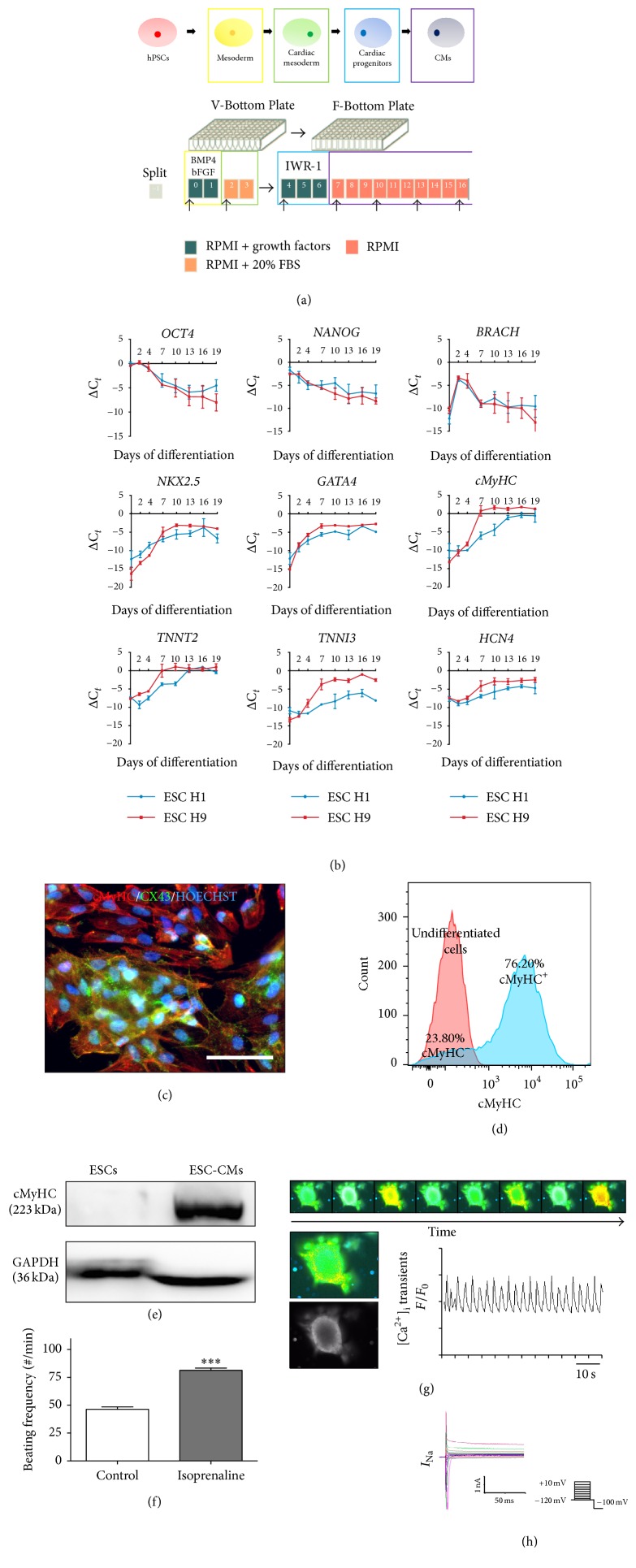
Characterization of the embryoid body- (EB-) based cardiac differentiation protocol. Characterization was performed on human embryonic stem cell-derived cardiomyocytes (ESC-CMs) without the addition of Activin A (ActA) during the early stage of differentiation (control condition). (a) Schematic representation of the EB-based cardiac differentiation protocol. (b) Expression profiles for pluripotency (*OCT4* and* NANOG*), mesodermal (*BRACH*), cardiac progenitor (*NKX2.5* and* GATA4*), and late-stage CM (*cMyHC*,* TNNT2*,* TNNI3*, and* HCN4*) markers, monitored during 19 days of differentiation. (c) IF analyses of dissociated EBs at day 20 of differentiation, showing areas of connected CMs (cMyHC; red) expressing connexin 43 (CX43; green). Nuclei were stained with Hoechst (blue). (d) Flow cytometry analyses of cMyHC^+^ CMs at day 20 of differentiation. Representative example of three independent experiments. (e) Western blot analyses quantifying the cMyHC protein levels of undifferentiated ESCs and CMs after 20 days of differentiation, normalized by GAPDH. (f) *β*-Adrenergic response of CMs to isoprenaline (1 *μ*M; isoproterenol), triggered at day 20 of differentiation. (g) Time course of intracellular Ca^2+^ handling at day 20 of differentiation, using the Ca^2+^-sensitive fluorescent indicator Fluo-4 (2.5 *μ*M), monitored by confocal microscopy. (h) Whole-cell Na^+^ recording, accessed by applying 500 ms voltage pulses to potentials between −120 and +10 mV in 5 mV increments from a holding potential of −100 mV at 0.5 Hz. RT-QPCR data are represented as ΔC_*t*_, normalized for the housekeeping genes* GAPDH*,* HPRT*, and* RPL13a*. Data are representative of three independent experiments and values are expressed as mean ± standard error of the mean (SEM). Significant difference is versus control and indicated as *P* < 0.001: ∗∗∗. Scale bar = 100 *μ*m.

The EB-based protocol, previously described by Burridge et al. [37–39], was used to differentiate the human ESC lines (H1 and H9) into functional CMs (Figure 1(a)).

One day before cardiac differentiation induction, human ESCs, cultured feeder-free on Matrigel, were dissociated into single cells with TrypLE (Thermo Fisher Scientific) at 37°C for 2 minutes and seeded in Matrigel-coated cell culture flasks at 1.0–1.5 × 10^5^ cells/cm^2^ in mTeSR medium supplemented with 10 *μ*M Rho-associated protein kinase (ROCK) inhibitor (Y-27632; VWR). At day 0 of differentiation, cells reached 80–90% of confluency and were dissociated with TrypLE and plated in V-bottom uncoated 96-well multiwell plates at a density of 5500 cells per well under hypoxia in a mesoderm induction medium containing RPMI 1640 (Thermo Fisher Scientific) with 1% chemically defined lipid concentrate (Thermo Fisher Scientific), 10 *μ*g/mL recombinant human insulin (Sigma-Aldrich), 450 *μ*M 1-Thioglycerol (Sigma-Aldrich), 2 mg/mL polyvinyl alcohol (PVA; Sigma-Aldrich), 0.1% Pen/Strep (Thermo Fisher Scientific), 1 *μ*M Y-27632, and the growth factors BMP4 (25 ng/mL), bFGF (5 ng/mL), and ActA (ranging from 0 to 100 ng/mL; all from PeproTech). PVA enhances EB formation and subsequently cardiac specification. At day 2, medium was changed with RPMI 1640 containing 20% foetal bovine serum (FBS; Thermo Fisher Scientific), 50 *μ*g/mL L-ascorbic acid 2-phosphate (Sigma-Aldrich) [40], 450 *μ*M 1-Thioglycerol, and 0.1% Pen/Strep and incubated for 48 h under normoxic conditions. At day 4, EBs were transferred to tissue culture-treated 96-well plates and incubated for 72 h with RPMI 1640 supplemented with 4 *μ*M of the Wnt inhibitor IWR-1 (Sigma-Aldrich). From day 10 onwards, medium without IWR-1 was used. In CRIPTO-1 interfering experiments, 5 *μ*M of the CFC1/CRIPTO-1 blocking peptide (CRIPTO-1 BP; GeneTex) was added to differentiating human ESCs during the first step of the differentiation protocol (from days 0 to 2). To validate the blocking efficiency of the CFC1/CRIPTO-1 BP in vitro, cells were treated with a CRIPTO-1 antibody (Thermo Fisher Scientific, PA1-16534; 10 *μ*M).

### 2.3. Quantitative Real-Time PCR

Total RNA was extracted using the PureLink RNA Mini Kit (Thermo Fisher Scientific) and treated with the DNA-Free Kit (Thermo Fisher Scientific) to assure highly pure RNA. 0.5 *μ*g RNA was reverse transcribed into cDNA with Superscript III Reverse Transcriptase First-Strand Synthesis SuperMix (Thermo Fisher Scientific). Quantitative real-time PCR was performed with the Platinum SYBR Green qPCR SuperMix-UDG (Thermo Fisher Scientific). The oligonucleotide primer sequences are listed in Supporting Information Table S1 (all from IDT). A 10-fold dilution series ranging from 10^−3^ to 10^−8^ of 50 ng/*μ*L human genomic DNA was used to evaluate the primer efficiency. Relative expression values were obtained by normalizing C_*t*_ values of the tested genes to C_*t*_ values of the housekeeping genes* GAPDH*,* HPRT*, and* RPL13a*.

### 2.4. Immunofluorescence Staining

EBs at day 7 in the differentiation process were stained for CM and early endodermal progenitor cell characterization. Cells were fixed with 4% paraformaldehyde (PFA; Polysciences) for 10 minutes at 4°C, permeabilized in PBS (Thermo Fisher Scientific) containing 0.2% Triton X-100 (Sigma-Aldrich) and 1% bovine serum albumin (BSA; Sigma-Aldrich) for 30 minutes, and blocked in 10% donkey serum (Sigma-Aldrich) for 30 minutes. Cells were incubated overnight at 4°C with the following primary antibodies: cardiac myosin heavy chain (cMyHC; Abcam BA-G5; 10 *μ*g/mL), connexin 43 (CX43; Santa Cruz H-150; 0.67 *μ*g/mL), and hepatocyte nuclear factor 4 alpha (HNF4*α*; Abcam, K9218; 5 *μ*g/mL), followed by the appropriate Alexa Fluor 488- and 594-conjugated secondary antibody (Thermo Fisher Scientific; 4 *μ*g/mL). Nuclei were stained with Hoechst (33342, Thermo Fisher Scientific; 1 : 3000) for 1 minute. Analyses were assessed using an Eclipse Ti Microscope and NIS-Elements AR 4.11 Software (both from Nikon).

### 2.5. Western Blot

Cells were lysed in RIPA buffer supplemented with 10 mM sodium fluoride, 0.5 mM sodium orthovanadate, 1 : 100 protease inhibitor cocktail, and 1 mM Phenylmethylsulfonyl Fluoride (PMSF). Equal amounts of protein (40 *μ*g) were heat-denatured in sample-loading buffer (50 mM Tris-HCl, pH 6.8, 100 mM DTT, 2% SDS, 0.1% bromophenol blue, and 10% glycerol), resolved by SDS-polyacrylamide gel electrophoresis, and then transferred to nitrocellulose membranes. The filters were blocked with Tris-buffered saline (TBS) containing 0.05% Tween and 5% nonfat dry milk and then incubated overnight at 4°C with the primary antibody cMyHC (Abcam, BA-G5; 2 *μ*g/mL) and normalized for GAPDH (Sigma-Aldrich G9545; 1 *μ*g/mL). Secondary horseradish peroxidase- (HRP-) conjugated antibody was diluted 1 : 5000 in TBS-Tween and 2.5% nonfat dry milk. After incubation with SuperSignal Dura Chemiluminescence substrate, the polypeptide bands were detected with GelDoc chemiluminescence detection system. Quantitation was performed on gels loaded and blotted in parallel and relative densitometry was obtained by normalizing the protein band versus background and housekeeping protein using the QuantityOne Software.

### 2.6. Flow Cytometry Analysis

Undifferentiated ESCs or EBs were harvested and dissociated with Collagenase IV for 20 minutes and StemPro Accutase Cell Dissociation Reagent (both from Thermo Fisher Scientific) for 3 minute at 37°C. To analyze the presence of CRIPTO-1 on HNF4*α*^+^ endodermal cells, EBs in early cardiac differentiation were dissociated, fixed with PFA for 10 minutes at 37°C, and permeabilized in ice-cold 90% methanol (Sigma-Aldrich) for 30 minutes on ice. Samples were stained for 1 hour at 37°C with the following antibodies: CRIPTO-1 (Thermo Fisher Scientific PA1-16534; 1 *μ*g/mL) and HNF4*α* (Abcam K9218; 1 *μ*g/mL), followed by the appropriate Alexa Fluor 488- and 647-conjugated secondary antibody (Thermo Fisher Scientific; 1 *μ*g/mL) for 30 minutes at 37°C. To determine the percentage of cMyHC^+^ CMs after cardiac differentiation without the addition of ActA, PFA fixed and methanol permeabilized EBs were stained for 1 hour at 37°C with the primary antibody cMyHC (Abcam, 3–48; 1.67 *μ*g/mL), followed by the appropriate Alexa Fluor 647-conjugated secondary antibody (Thermo Fisher Scientific; 1 *μ*g/mL) for 30 minutes at 37°C. To evaluate a possible ActA additional proliferative effect on EBs in early cardiac differentiation, Ki-67 and 7-AAD (7-Aminoactinomycin D) staining assay were performed. PFA fixed and methanol permeabilized EBs were stained for 20 minutes at RT with the primary antibody Ki-67 (BD Pharmingen, B56; 1 *μ*g/mL), followed by the Alexa Fluor 647-conjugated secondary antibody (Thermo Fisher Scientific; 1 *μ*g/mL) for 20 minutes at RT. The 7-AAD staining (eBioscience; 1 : 25) was performed with unfixed cells, incubated for 5 minutes at RT. Hanks' Balanced Salt Solution (HBSS; Thermo Fisher Scientific) with CaCl_2_ and MgCl_2_ supplemented with 2% FBS, 10 mM HEPES, and 10 mM NaN_3_ (both from Sigma-Aldrich; pH 7.2) was used as staining buffer. Cells were analyzed and quantified by flow cytometry (BD FACSCanto I or II with HTS; BD Biosciences) and FlowJo Software (FlowJo LLC).

### 2.7. Patch Clamp Electrophysiology and Ca^2+^ Measurements

Single cells were seeded on Matrigel-coated coverslips for whole-cell patch clamp and Ca^2+^ recordings. Cells were perfused with a solution containing the following (in mM): 137 NaCl, 5.4 KCl, 1.8 CaCl_2_, 0.5 MgCl_2_, 10 glucose, and 10 Na-HEPES; pH was adjusted to 7.4 with NaOH. The patch clamp pipettes were filled with a solution containing the following (in mM): 120 K-Asp, 20 KCl, 10 HEPES, 5 Mg-ATP, 10 NaCl, and 0.05 K_5_Fluo-4; pH was adjusted to 7.2 with KOH.

APs were measured during whole-cell current clamp with an EPC-9 amplifier (HEKA Elektronik) at a sampling rate of 10 kHz. Patch electrode resistances were between 2.5 and 3 MΩ when filled with intracellular solution. Capacitance and access resistance were monitored continuously. Experiments were performed at 37°C. APs were recorded after a 5 ms pulse of 300 pA, at a 1 Hz frequency. Signals were filtered with 2.9 kHz and 10 KHz Bessel filters. Data were analyzed with Clampfit.

Simultaneously with the patch clamp recordings, 2D images acquisition of the Fluo-4 signal was recorded using a confocal microscope (Nikon A1R, resonant mode, 40x 1.3 NA oil immersion objective) at a scan speed of 1 image each 110 ms (average of 4 images). Data were analyzed using ImageJ by measuring the background subtracted mean fluorescence intensity (MFI) of the cells, excluding the nucleus area, over time.

### 2.8. Statistical Analysis

Data were statistically analyzed using GraphPad Prism. All data were reported as mean ± standard error of the mean (SEM). Differences between groups were examined for statistical significance using a two-tailed unpaired Student's* t*-test (with Welch's correction if necessary), Fisher's test or ANOVA. Significance of the differences was indicated as follows: *P* < 0.05: *∗* versus control, § versus 10 ng/mL ActA, # versus 25 ng/mL ActA, and + versus 50 ng/mL ActA; *P* < 0.01: *∗∗* versus control, §§ versus 10 ng/mL ActA, ## versus 25 ng/mL ActA, and ++ versus 50 ng/mL ActA; and *P* < 0.001: ∗∗∗ versus control, §§§ versus 10 ng/mL ActA, ### versus 25 ng/mL ActA, and +++ versus 50 ng/mL ActA.

## 3. Results

### 3.1. Activin A Dose-Dependent Regulation of Pluripotency and Lineage Markers during Early Cardiomyocyte Differentiation

**Figure 2:**
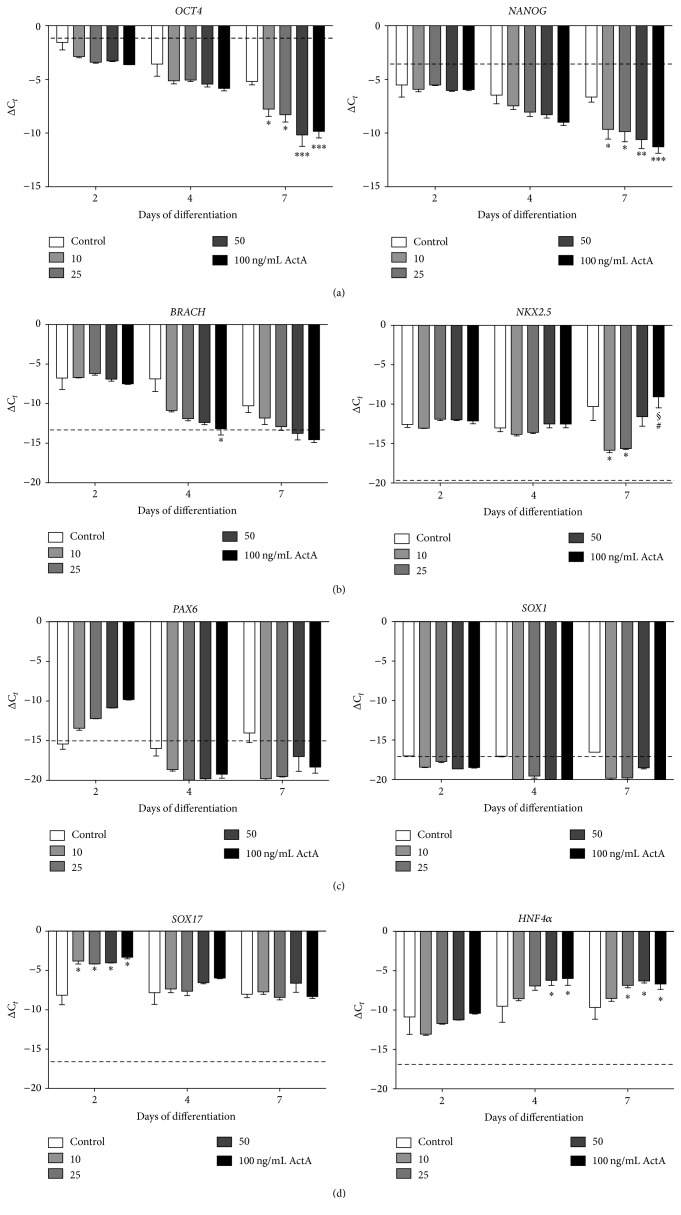
Gene expression analyses of human embryonic stem cells (ESCs) subjected to cardiac differentiation in the presence of Activin A (ActA). Expression profiles of human ESC-derived CMs (ESC-CMs) for (a) pluripotency (*OCT4, NANOG*), (b) mesodermal (*BRACH, NKX2.5*), (c) ectodermal (*PAX6, SOX1*), and (d) endodermal (*SOX17, HNF4α*) lineage markers, monitored at days 0, 2, 4, and 7 of differentiation. Dashed lines show basal expression levels of undifferentiated ESCs. Each data point is represented as ΔC_*t*_, normalized for the housekeeping genes* GAPDH*,* HPRT*, and* RPL13a*. Note that ActA treatment during the early phase of cardiomyocyte (CM) differentiation directed human ESCs towards the mesendoderm cell fate. Data are representative of three independent experiments and values are expressed as mean ± standard error of the mean (SEM). Significant differences are indicated as *P* < 0.05: *∗* versus control, $ versus 10 ng/mL ActA, and # versus 25 ng/mL ActA; *P* < 0.01: *∗∗* versus control; *P* < 0.001: ∗∗∗ versus control.

The human ESC lines H1 and H9 were differentiated towards CMs, according to the protocol described (see Materials and Methods; Figures 1(a)–1(h)). During the early phase of cardiac differentiation, human ESCs in EB aggregates were treated with different concentrations of ActA, ranging from 0 to 100 ng/mL. Addition of 10, 25, 50, or 100 ng/mL ActA resulted in a more pronounced downregulation of the pluripotency markers* OCT4* and* NANOG* (Figure 2(a)). To investigate the effect of ActA on the early cell fate during in vitro cardiac differentiation, the expression of genes corresponding to the three developmental lineages was determined. Pretreatment with ActA did not increase the expression of the mesodermal marker* BRACH* on day 2 of differentiation, although a significant downregulation was observed at day 4 in the presence of 100 ng/mL ActA compared to the untreated condition (0 ng/mL ActA; Figure 2(b),* left panel*). Moreover, the cardiac progenitor marker* NKX2.5* was significantly upregulated on day 7 in human ESC-CMs treated with 100 ng/mL ActA (Figure 2(b),* right panel*). As expected, ActA treatment did not direct the differentiation process towards the ectodermal lineage, although a transient upregulation of* PAX6* was observed in response to ActA (Figure 2(c)). Interestingly, ActA significantly increased early and intermediate endodermal markers, respectively,* SOX17* (Figure 2(d),* left panel*) and* HNF4α* (Figure 2(d),* right panel*). Thus, addition of ActA during the initial step of differentiation induced a faster and stronger decrease in the pluripotency and promoted a mesendodermal cell fate, significantly shown by the expression of* BRACH* and* SOX17*. A concentration of 100 ng/mL ActA was the most favourable for efficient cardiac progenitor cell induction via mesendoderm.

### 3.2. Activin A Promoted Induction of Cardiomyocytes

**Figure 3:**
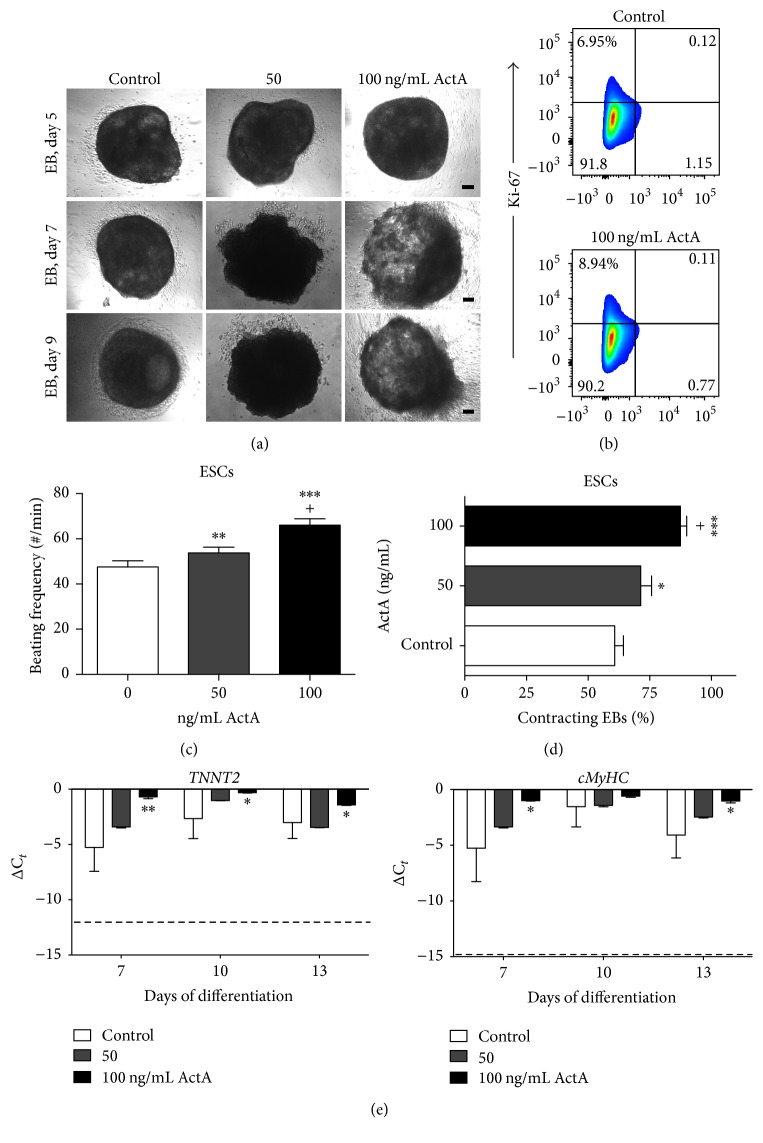
ActA increased human ESC-CM differentiation efficiency. High doses of 50 and 100 ng/mL ActA promoted CM differentiation. (a) Brightfield images of EB morphology at days 5, 7, and 9 of differentiation of ActA treatment conditions (50 and 100 ng/mL) compared to the untreated cells (0 ng/mL ActA). (b) Proliferation was assessed using Ki-67, showing that 6.95% of the control cells and 8.94% of the ActA treated cells were in a proliferative stage. Flow cytometry analysis is a representative example of three independent experiments. (c) Quantification of contracting rate (beating frequency EBs per minute) and (d) percentage of contracting EBs at day 10 of differentiation. Efficiency is expressed as percentage of wells containing beating EBs to the total number of wells. (e) Gene expression analyses of late-stage CM genes (*TNNT2, cMyHC*) during 13 days of differentiation. Dashed lines show basal expression levels of undifferentiated ESCs. Each data point is represented as ΔC_*t*_, normalized for the housekeeping genes* GAPDH*,* HPRT*, and* RPL13a.* Data are representative of three independent experiments and expressed as mean ± SEM. Significant differences are indicated as *P* < 0.05: *∗* versus control and + versus 50 ng/mL; *P* < 0.01: *∗∗* versus control; *P* < 0.001: ∗∗∗ versus control. Scale bar = 100 *μ*m.

To investigate whether a high dose of ActA could support further differentiation into CMs, EB morphology of human ESC-CMs at days 4, 5, 7, and 9 of differentiation was evaluated after a treatment with 50 and 100 ng/mL ActA. At day 4, EBs exhibited a comparable appearance irrespective of the ActA concentrations applied (data not shown). At days 5, 7, and 9, round shaped EBs were formed in the control condition and when high concentrations of ActA (50 or 100 ng/mL) were added to the mesoderm induction medium (Figure 3(a)). In the control and high dose ActA conditions, EBs contained a significant surface area of contracting CMs (Supplemental Online Videos 1A and 1D-E, in Supplementary Material available online at https://doi.org/10.1155/2017/4651238). No significant additional proliferative effect of ActA was detected at day 2 of EB-based cardiac differentiation (Figure 3(b)). The mean beating frequency of the EBs treated with 50 and 100 ng/mL ActA was, respectively, 54 (53.8 ± 2.49) and 66 (66.0 ± 2.92) beats/min, while 48 (47.6 ± 2.70) beats/min were registered for the control (Figure 3(c)). Spontaneous beating EBs were observed at day 10 of differentiation. The percentage of contracting human ESC-CM EBs was 61% (60.8 ± 15.83) for control conditions and increased to, respectively, 71% (71.4 ± 12.36) and 88% (87.5 ± 11.34) for EBs treated with 50 and 100 ng/mL ActA, resulting in a remarkable increase of contracting EBs (Figure 3(d)). To confirm the effect of ActA on the CM differentiation efficiency, we analyzed the gene expression profiles of the late-stage CM markers* TNNT2* and* cMyHC*. On day 7 of differentiation, the expression of* TNNT2* and* cMyHC* was upregulated in EBs treated with 50 or 100 ng/mL ActA compared to the control condition (Figure 3(e)). Thus, the in vitro CM differentiation was more efficient with high doses of ActA (50 and 100 ng/mL), resulting in higher early CM maturation.

### 3.3. Activin A Increased HNF4*α*^+^ Cell Population during Cardiac Differentiation

**Figure 4:**
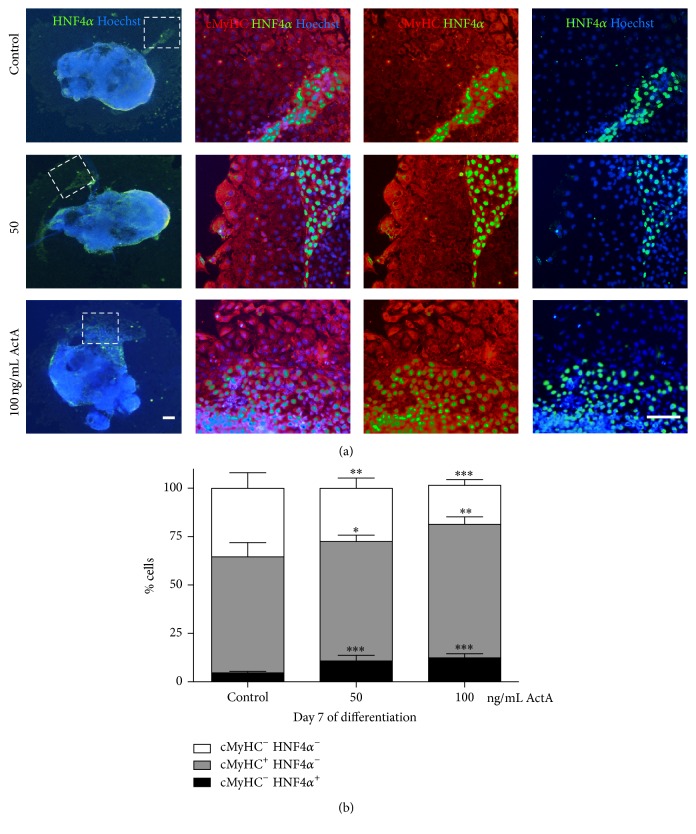
ActA promoted CM differentiation by inducing HNF4*α*^+^ endoderm-like cell population. High doses of 50 and 100 ng/mL ActA during the early phases of cardiac differentiation increased the amount of HNF4*α*^+^ endoderm-like cells, which subsequently promoted the CM differentiation efficiency. (a) IF analyses for HNF4*α* (green), cMyHC (red), and Hoechst (blue) in 7-day-old EBs for control and 50 and 100 ng/mL ActA treated conditions. Dashed squares indicate the region of interest, corresponding to IF pictures at higher magnification. (b) Quantifications of cMyHC^+^, HNF4*α*^+^ and cMyHC^−^ HNF4*α*^−^ cell populations in the presence (50 and 100 ng/mL) or absence of ActA are reported as percentages of control conditions. Data are representative of three independent experiments and expressed as mean ± SEM. Significant differences are versus control and indicated as *P* < 0.05: *∗*; *P* < 0.01: *∗∗*; and *P* < 0.001: ∗∗∗. Scale bar = 100 *μ*m.

In line with the previously described* HNF4α* gene expression analyses during ActA-induced cardiac differentiation (Figure 2(d),* right panel*), immunostaining demonstrated that HNF4*α*^+^ endoderm-like cells were present and, moreover, in close proximity of the cMyHC^+^ CMs in 7-day-old EBs in control and 50 and 100 ng/mL ActA treated conditions (Figure 4(a)). ActA promoted the differentiation towards a HNF4*α*^+^ endoderm-like cell population via mesendoderm induction, as demonstrated by the quantification of HNF4*α*^+^ nuclei that increased to 18% (17.5 ± 3.92) and 19% (19.0 ± 1.43) in, respectively, the 50 and 100 ng/mL ActA treated conditions compared with 9% (9.2 ± 2.15) reported in the control (Figure 4(b)). ActA increased also the percentage of cMyHC^+^ cells, 62% (61.6 ± 6.25) in the 50 ng/mL and 69% (68.9 ± 6.80) in the 100 ng/mL ActA treated cells compared to 49% (49.2 ± 14.05) in the control condition (Figure 4(b)). Thus, addition of high concentrations of ActA increased the percentage of cMyHC^+^ CMs, confirming an increased HNF4*α*^+^ endoderm-like cell generation mediated by ActA.

### 3.4. Antagonizing CRIPTO-1 Signaling Impaired Cardiomyocyte Differentiation

**Figure 5:**
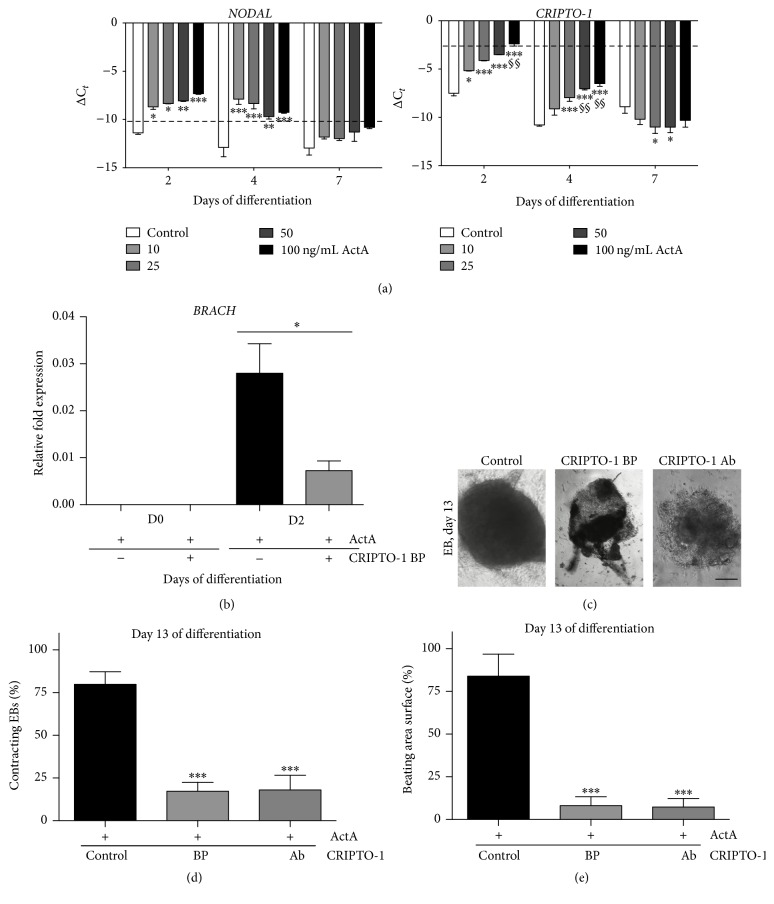
Human ESC-CM differentiation potential after CRIPTO-1 interference. CRIPTO-1 blocking peptide (BP) impaired ActA-directed in vitro cardiac differentiation. (a) Gene expression of the TGF-*β* family members* NODAL* and* CRIPTO-1* during a 13-day differentiation period. Dashed lines show basal expression levels of undifferentiated ESCs. (b) Normalized fold change of the mesodermal marker* BRACH*, measured at day 2 of differentiation after CRIPTO-1 BP treatment. Data, normalized for the housekeeping genes* GAPDH*,* HPRT*, and* RPL13a*, are representative of three independent experiments and expressed as mean ± SEM. (c) Brightfield pictures of ESC-CMs at day 13 of differentiation after CRIPTO-1 BP treatment or after addition of a human CRIPTO-1 antibody (Ab). (d) Percentage of contracting EBs and of (e) beating area surface in differentiated CMs pretreated with 100 ng/mL ActA and 5 *µ*M CRIPTO-1 BP or pretreated with 100 ng/mL ActA and a human CRIPTO-1 Ab (10 *µ*M). Data, normalized for the housekeeping genes* GAPDH*,* HPRT*, and* RPL13a*, are representative of four independent experiments and expressed as mean ± SEM. Significant differences are indicated as *P* < 0.05: *∗* versus control; *P* < 0.01: *∗∗* versus control and §§ versus 10 ng/mL ActA; *P* < 0.001: ∗∗∗ versus control. Scale bar = 200 *μ*m.

To understand the influence of an ActA addition on the expression of members of the TGF-*β* signaling pathway during our ESC EB-based cardiac differentiation in a human setting, gene expression analyses were performed for* NODAL*,* CRIPTO-1,* and the transmembrane types I (*ALK4* or* ACVR1B*) and II (*ACVR2A* and* ACVR2B*) activin receptors. No significant differences were observed in the expression levels of* ALK4*,* ACVR2A,* and* ACVR2B* in response to the different ActA concentrations (Supporting Information Fig. 1). However, the expression of* NODAL* and* CRIPTO-1* transcripts was significantly increased in an ActA dose-dependent way at days 2 and 4 (no ActA dose-dependent effect observed at day 7), suggesting that both signaling molecules were closely related (Figure 5(a),* left* and* right panel*). In undifferentiated human ESCs,* CRIPTO-1* gene expression has been shown to be high (Figure 5(a),* right panel*, dashed line). During differentiation,* CRIPTO-1* expression decreased significantly in the control condition starting from day 2. Human ESCs treated with different ActA concentrations showed a dose-dependent increase in* CRIPTO-1* levels observed on day 2 compared to controls. When treated with 100 ng/mL ActA,* CRIPTO-1* transcripts on day 2 were maintained at expression levels found in undifferentiated human ESCs. From day 4 onwards,* CRIPTO-1* content decreased drastically in all conditions (Figure 5(a),* right panel*). To confirm the importance of CRIPTO-1 during cardiac differentiation, the CFC1/CRIPTO-1 blocking peptide (CRIPTO-1 BP) was added from day 0 until day 2 of differentiation in combination with 100 ng/mL ActA (Supporting Information Fig. 2). Addition of CRIPTO-1 BP resulted in a significantly decreased* BRACH* transcript level on day 2 (Figure 5(b)). Moreover, we observed impairment in the EB structure in the presence of CRIPTO-1 BP (Figure 5(c) and Supplemental Online Video 2A-B). The percentage of beating EBs was evaluated on day 13. CRIPTO-1 BP significantly reduced the percentage of beating EBs from 80% (79.9 ± 2.60) to 17% (17.3 ± 1.85) (Figure 5(d)). In addition, the surface of the beating areas was decreased in the presence of CRIPTO-1 BP (83.9 ± 3.45 versus 8.1 ± 1.41) (Figure 5(e)). Thus, high concentrations of ActA maintained high levels of* CRIPTO-1* expression during the early phase of cardiac differentiation. Blocking the CRIPTO-1 signaling pathway impaired cardiac differentiation in vitro.

### 3.5. CRIPTO-1 Was Expressed by Activin A-Induced HNF4*α*^+^ Endoderm-Like Cells

**Figure 6:**
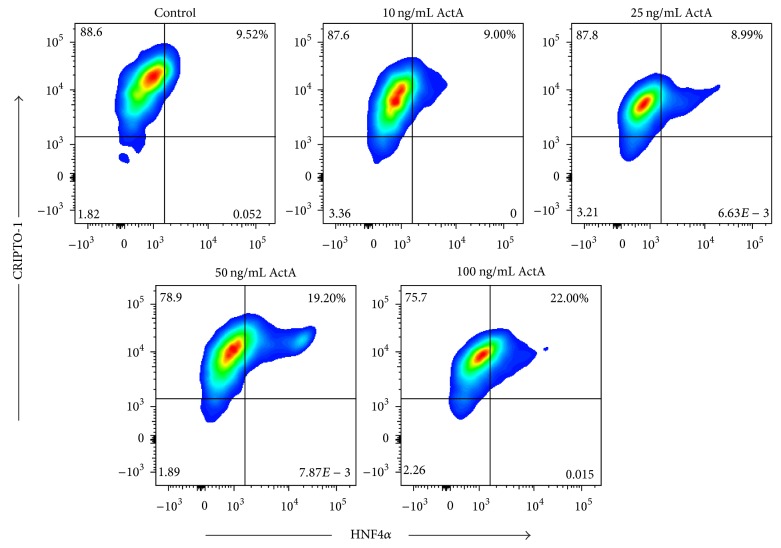
CRIPTO-1 and HNF4*α* expression profiles in ActA treated human ESCs during early cardiac differentiation. HNF4*α*^+^ endodermal-like cells had a higher expression level of CRIPTO-1 in the control and 50 and 100 ng/mL ActA condition compared to the 10 and 25 ng/mL ActA treatment. Flow cytometry analyses of HNF4*α* and CRIPTO-1 expression after induction of human ESC-CM differentiation with the indicated concentrations of ActA. Representative example of three independent experiments.

It has already been shown that Cripto-1 was highly present in murine mesendoderm cells [32, 33] and that it could promote CM differentiation [31, 34]. However, in the human setting, the cell fraction that expressed CRIPTO-1 was still unknown. We hypothesized that HNF4*α*^+^ endoderm-like cells, induced by ActA, might express the membrane-bound isoform of CRIPTO-1. As a consequence, HNF4*α*^+^ cells could be responsible for an improved CM differentiation efficiency through a CRIPTO-1-mediated mechanism. By flow cytometric analyses, undifferentiated human ESCs stained positive for CRIPTO-1 and negative for HNF4*α* (Supporting Information Fig. 3A-B). The CRIPTO-1^+^ HNF4*α*^+^ cell population was quantified at day 3 of cardiac differentiation with a percentage that varied in an ActA concentration-dependent manner (Figure 6). In the control condition, 9.52% (±1.58) of the differentiating cells were double positive for CRIPTO-1 and HNF4*α* (*first panel*). In the 10 and 25 ng/mL ActA condition, the number of double positive cells is similar to controls (*second and third panel*). However, the percentage of CRIPTO-1^+^ HNF4*α*^+^ cells increased at high doses of ActA, respectively, 19.20% (±0.95) for 50 ng/mL ActA and 22.00% (±3.25) for 100 ng/mL ActA (*fourth and fifth panel*). These results were in consonance with the immunostaining data for HNF4*α* quantification (Figure 4(b)). These data demonstrated that a high dose of 50 and 100 ng/mL ActA increased the percentage of HNF4*α*^+^ endoderm-like cell population expressing the surface-bound fraction of CRIPTO-1.

### 3.6. Activin A Treatment during Early Cardiac Differentiation Supported Maturation towards Cardiomyocytes

**Figure 7:**
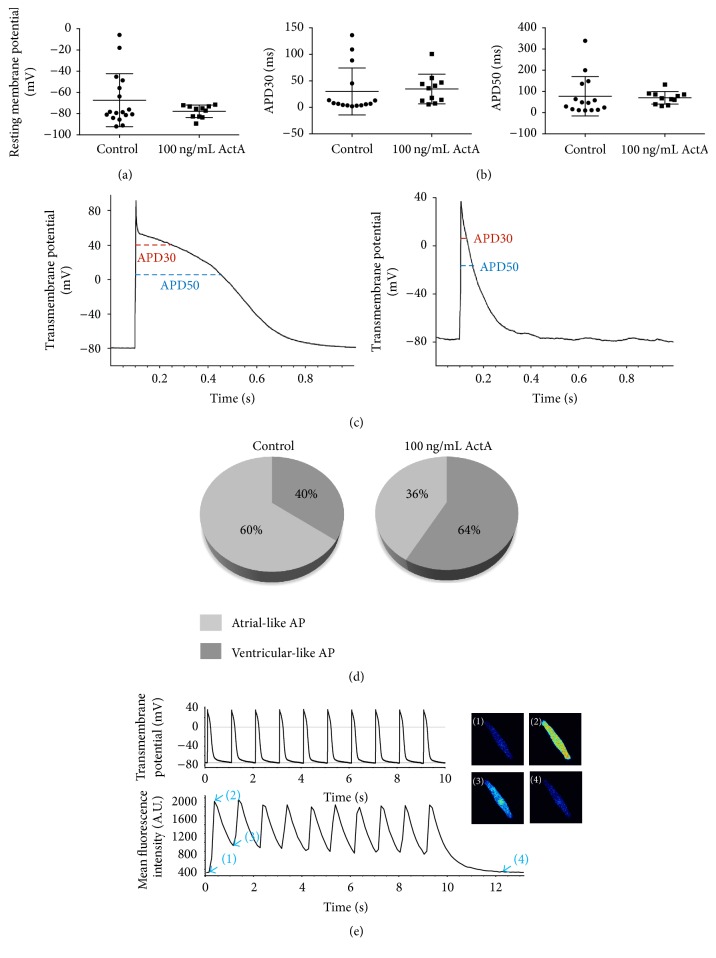
Phenotyping CM differentiation showed both atrial- and ventricular-like (action potentials) APs. (a) Resting membrane potential (RMP) of human ESCs-CMs, measured during current clamp (whole-cell patch clamp recording) with or without a pretreatment of 100 ng/mL ActA. Each data point is a single cell measurement. (b) AP duration at 30% and 50% repolarization (APD30 and APD50). (c) Example of AP from a typical ventricular-like cell (*left panel*) and an atrial-like cell (*right panel*). (d) Percentages of CMs exhibiting an atrial-like or ventricular-like AP phenotype with and without ActA addition. (e) Simultaneous intracellular Ca^2+^ measurements using Fluo-4 and current clamp recording in a human ESC differentiated into CMs.

For the cardiac differentiation, we used 25 ng/mL BMP4 and 5 ng/mL bFGF to initiate mesoderm induction. To induce NKX2.5^+^ cardiac progenitor cells, the IWR-1 Wnt signaling pathway inhibitor was added on day 4 (Supporting Information Fig. 4A-C). IWR-1 has been shown to direct human ESC-CM differentiation towards an atrial-like phenotype [41]. To study the CM subtype specification in response to ActA, APs of dissociated EB-CMs were analyzed at day 25 of differentiation using the patch clamp technique (Figures 7(a)–7(c)). Firstly, we measured the resting membrane potential (RMP) (Figure 7(a)). Cells treated with ActA (*n* = 11) were well polarized with a RMP of −70 mV or more negative, whereas the RMP for cells without ActA (*n* = 17) showed a more heterogeneous distribution. These data suggested a lower degree of maturation without ActA. To study AP characteristics, cells with a low RMP (i.e., more positive than −40 mV, *n* = 2) were excluded. Then, the APD30 and APD50 (30% and 50% repolarization) for early repolarization properties of the APs were measured (Figure 7(b),* right* and* left panel*). In the absence of ActA, early repolarization (low APD30) appeared to be common (11/15 cells had an APD30 lower than 15 ms versus 4/11 cells with ActA). Moreover, this could also be observed for APD50 where 8 cells had an APD50 lower than 30 ms without ActA and none was with ActA. This indicated that there was a trend that cells had a more pronounced plateau phase in the presence of ActA. According to the overall profile and the previously measured properties (APD30, APD50), we categorized the cells into an atrial-like or a ventricular-like phenotype (Figures 7(c)–7(d)), without ActA 9/15 cells had an atrial-like profile versus 4/11 cells with ActA (Fisher's test = NS). However, it is important to note that there was a spectrum of APs from an atrial-like to a ventricular-like phenotype, as recently described in human iPSC-CMs [42]. After 40 days in culture, we could also observe cytosolic Ca^2+^ transients accompanying the stimulated APs (Figure 6(e) and Supplemental Online Video 3). Taking these data together, the protocol used here for cardiac differentiation (with and without 100 ng/mL ActA) allowed production of atrial- and ventricular-like CMs associated with AP and Ca^2+^ transients, showing a trend for a more homogeneous phenotype and polarization with ActA.

## 4. Discussion

Human ESCs exhibit the potential to generate functional cell types that can be used as renewable cell source to study embryogenesis and, consequently, gain better insights in signaling pathways involved in PSC-driven differentiation, including lineage specification, lineage commitment, and terminal differentiation.

In the current study we analyzed the role of ActA on the early formation of primitive streak and mesoderm/endoderm from human ESCs during cardiac differentiation. We demonstrated, in the here described cardiac differentiation protocol, high concentrations of ActA during the initial steps of differentiation fated human ESCs towards CMs with a prevalent ventricular-like AP phenotype and with AP and Ca^2+^ handling properties similar to mature cardiac myocytes. To study the intercellular interactions between mesodermal and endodermal cell populations, an EB-based cardiac differentiation system was applied, representing better the three-dimensional (3D) environment of human cardiac development. A clinically more relevant EB-based serum-free protocol has been described as alternative for our protocol, though less efficient and therefore less suitable for mesodermal/endodermal interaction studies [38]. For translational purposes, a monolayer in serum-free conditions would be the most pertinent protocol to generate human ESC-derived ventricular CMs to develop a platform for pharmacological screening or to study heart diseases.

Several studies have been conducted to explore the interplay between the TGF-*β*/ActA/NODAL, bFGF, and BMP signaling pathways on early cell fate determination. However, conflicting results exist regarding the effects of these pathways in mouse and human ESCs. Addition of ActA alone has been shown to maintain self-renewal properties [43] and ActA/NODAL together with bFGF signaling coordinately maintained pluripotency in human ESCs [44]. Studies in mouse ESCs indicated that either BMP4 or ActA alone or in combination induced primitive streak markers, even if BMP4 seemed to exert a dominant effect [45, 46]. Recently, Fiorenzano et al. have reported an early function of* CRIPTO-1* in the very first lineage decision made by the early embryo. CRIPTO-1 was one of the earliest epiblast markers and played a pivotal functional role in the acquisition and maintenance of mouse and human pluripotency. Moreover, they demonstrated that CRIPTO-1 was required to generate and sustain bFGF/ActA EpiSCs, and a CRIPTO-1 deficiency skewed ESC differentiation towards the trophoblast lineage in vitro [35].

Inhibition of ActA signaling blocked mesendoderm induction in BMP4 treated mouse ESCs, while BMP4 signaling inhibition had no impact on the mesendodermal cell fraction observed in in vitro cultures treated with ActA, thereby pointing out the pivotal role of ActA/Nodal signaling in mesendoderm formation. Moreover, ActA induced higher levels of the anterior mesendoderm/early endoderm markers,* Foxa2* and* Sox17*, while BMP4 slightly upregulated these genes [46]. Similarly, mouse ESCs treated with high levels of ActA differentiated in a more anterior streak fate, including cardiac mesoderm and definitive endoderm [14]. For human PSCs, there was evidence that a short-term BMP4 treatment of human ESCs promoted mesoderm differentiation [47]. There was also evidence that although ActA signaling was permissive for differentiation of human ESCs towards mesendodermal cell fate, it was not sufficient. However, human PSC differentiation to mesoderm, and subsequently CMs, was more robust when a combination of BMP4/ActA [21] or a combination of BMP4, bFGF, and ActA was used [20]. Interestingly, one study reported that differentiation of human ESCs with BMP4 but without ActA resulted in a decreased survival rate and decreased efficiency in generating mesendoderm and/or mesoderm [47]. Consistent with these studies, we demonstrated that the addition of ActA in combination with BMP4 and bFGF increased the expression of the early endodermal marker* SOX17* and the definitive endodermal marker* HNF4α* in human ESCs. More recently, a monolayer-based cardiac differentiation protocol has been published, applying the Wnt signaling agonist CHIR in combination with high or low levels of ActA [48]. This study is not in contrast with our data. In fact, consistent with our results the authors showed that coinduction with CHIR and high ActA levels induced definitive endoderm, whereas CHIR supplemented with low ActA levels improved cardiomyogenic efficiency. By contrast, CHIR plus low levels of BMP4 consistently and strongly inhibited cardiomyogenesis. With our work, we extended this study and provided novel mechanistic insights. We identified an upregulation of the endodermal genes* SOX17* and* HNF4α* associated with increased CRIPTO-1 signaling when ActA was added during early CM differentiation.

The ActA/NODAL/CRIPTO-1 signaling plays an important role in maintaining human ESCs in an undifferentiated pluripotency state [49, 50]. Several previously published papers have indicated Cripto-1 as a critical player in mouse cardiomyogenesis. Similarly to our CRIPTO-1 blocking experiments, Cripto-1 knockout mouse ESCs lacked the ability to generate beating CMs in vitro [31]. Minchiotti et al. demonstrated that the Nodal/Cripto-1 pathway was involved in mouse ESC differentiation towards functional beating CMs, while preventing neuroectoderm differentiation [33, 50, 51]. Moreover, mesendodermal cells highly expressed Cripto-1 [32, 33]. The dynamics of Cripto-1 signaling, including timing, strength, and duration of the signal, have been described to be critical for correct specification and differentiation of the cardiac lineage. Cripto-1 signaling needed to be initiated in an early acting window of time, and the transient presence of Cripto-1 was inadequate, while sustained Cripto-1 signaling (at least 24 hours) was required to promote cardiomyogenesis [33]. Another study reported that hypoxia-mediated murine ESC differentiation to mesendoderm formation, and subsequently CM differentiation, occurred via a Cripto-1-mediated signaling pathway [34]. However, none of these studies succeeded in defining a human origin for CRIPTO-1 expression. In our study, we confirmed that the addition of ActA, in a dose-dependent manner, increased* CRIPTO-1* transcripts in the mixed EB progeny. We showed for the first time that, in an EB-based differentiation of human ESC towards CMs, CRIPTO-1 induction was required to induce differentiation towards CMs. As CRIPTO-1 existed either as a cell membrane or as secreted isoform, precise examination of the cell types expressing CRIPTO-1 was difficult. We observed that the addition of ActA resulted in a significantly greater population of HNF4*α*^+^ endodermal cells in the EB outgrowth. Our data has shown that, within ActA exposed EBs, the HNF4*α*^+^ endoderm-like cell population expressed CRIPTO-1 and that CRIPTO-1 expression in the HNF4*α*^+^ cells was maintained at levels found in undifferentiated human ESC at day 2 of differentiation. Therefore, we suggested that HNF4*α*^+^ cells could be important contributors of the increased human CM differentiation via a CRIPTO-1 signaling mechanism.

Consistent with our results, Mummery et al. have already identified visceral endoderm cells as a source of molecular cues that resulted in human ESCs differentiating to CMs [4]. Further studies will be useful to understand if HNF4*α*^+^ cells were the original visceral endoderm inducers to promote CM differentiation and eventually maturation in order to build up robust CM differentiation protocols from human ESCs.

The inclusion of high levels of insulin during the early stage of cardiac differentiation has been shown to induce strong inhibitory effects on cardiomyogenesis [52]. Indeed, in a more recent paper, insulin has been shown to inhibit cardiac mesoderm, but not mesendoderm formation, during cardiac differentiation induced by using TGF-*β* superfamily ligands. However, the authors claimed that the modulation of the canonical Wnt signaling pathway can rescue this insulin-mediated inhibition [53]. We confirmed the release of the insulin inhibitory effect by adding the Wnt inhibitor IWR-1 during the cardiac progenitor stage.

Human PSC-CMs offer a potentially relevant model system for drug discovery. Despite their suitability for cardiotoxicity testing and safety pharmacology, their application in validating novel drug candidates for atrial- or ventricular-specific heart disorders requires in vitro cultures enriched in atrial- or ventricular-like CMs. Currently, cardiac differentiation protocols of human PSCs result usually in a heterogeneous pool of atrial-, ventricular-, and nodal-like CMs [3]. A recently published paper has reported the importance of the exogenous addition of retinoic acid (RA) during human ESC differentiation for atrial chamber cell development [54]. Treating differentiating human ESCs with the Wnt inhibitors, IWP-4 and IWR-1, resulted in CMs with differing atrial and ventricular expression levels of, respectively,* MLC-2a* and* MLC-2v* [55]. Inhibition of the Wnt signaling pathway by using IWR-1 has been shown to differentiate human ESC-CMs towards mostly atrial-like CMs [41]. However, conflicting data exist [56]. Human ESCs, subjected to the protocol described in this study with ActA, were well polarized and with a trend towards a predominant ventricular AP phenotype. Further studies will examine the persistence and further development of this phenotype at later time points. Interestingly, cells treated with ActA exhibited a RMP of −70 mV or more negative, whereas the RMP for cells without ActA treatment showed a more heterogeneous distribution, suggesting a lower degree of CM maturation in the absence of ActA.

In conclusion, high concentrations of ActA during the initial phase of EB-based cardiac differentiation enhanced the definitive endodermal cell population, which in turn promoted differentiation of human ESCs towards CMs, and maturation in early stages of differentiation, improving the beating frequency and the percentage of contracting EBs.

## Supplementary Material

Supplementary Video 1: Embryoid body (EB) formation and morphology of human embryonic stem cells (ESCs) during in vitro cardiac differentiation. High doses of ActA (50 and 100 ng/mL ActA) increased beating frequency and contracting area of EBs. Representation of EBs at day 10 of cardiac differentiation of human ESCs (A) under control conditions (without ActA), with (B) 10 ng /mL ActA, (C) 25 ng/mL ActA, (D) 50 ng/mL ActA, and (E) 100 ng/mL ActA. Contracting areas are indicated by white dashed lines.
