# Supplementary material for: Activin A Modulates CRIPTO-1/HNF4α+ Cells to Guide Cardiac Differentiation from Human Embryonic Stem Cells
Source: Stem Cells Int. 2017 Jan 9;2017:4651238. doi: 10.1155/2017/4651238 (PMC5253508; doi:10.1155/2017/4651238)
Supplement: Supplementary file 1 — Supplementary Video 1: Embryoid body (EB) formation and morphology of human embryonic stem cells (ESCs) during in vitro cardiac differentiation. High doses of ActA (50 and 100 ng/mL ActA) increased beating frequency and contracting area of EBs. Representation of EBs at day 10 of cardiac differentiation of human ESCs (A) under control conditions (without ActA), with (B) 10 ng /mL ActA, (C) 25 ng/mL ActA, (D) 50 ng/mL ActA, and (E) 100 ng/mL ActA. Contracting areas are indicated by white dashed lines. [file 4651238.f1.zip › duelen et al. - supplementary figure legends_sci_1816894.pdf]

Supporting Information Figure Legends

Supplementary Figure 1: **Gene expression analyses of human ESCs after ActA treatment during *in vitro* cardiac differentiation.** No significant differences were observed for the expression of the transmembrane type I (*ALK4* or *ACVR1B*) and II (*ACVR2A* and *ACVR2B*) activin receptors in response to the different ActA concentrations. Expression profiles of human ESC-CMs for *ALK4* or *ACVR1B*, *ACVR2A*, *ACVR2B* and *ActA*, monitored at day 0, 2, 4 and 7 of differentiation. Dashed lines show basal expression levels of undifferentiated ESCs. Each data point is represented as ΔCt, normalized for the housekeeping genes *GAPDH, HPRT* and *RPL13a*. Data are representative of three independent experiments and values are expressed as mean ± SEM. Significant differences are vs. control and indicated as $P < 0.05$: *; $P < 0.01$: **; and $P < 0.001$: ***.

Supplementary Figure 2: **Dose-cytotoxicity analyses for CRIPTO-1 blocking peptide (BP).** Addition of 5 μM CRIPTO-1 BP did not induced a significant amount of cell death during the early phase of cardiac differentiation. Cell death was determined by flow cytometry using the live/cell death staining 7-AAD (7-Aminoactinomycin D), showing that 8.68% of the cells died during the procedure. 17.60% of the cells died when treated with 5 μM CRIPTO-1 BP, and respectively 19.70% and 22.10% died during the treatment of 15 μM and 45 μM CRIPTO-1 BP. Flow cytometry analysis is a representative example of three independent experiments.

Supplementary Figure 3: **CRIPTO-1 and HNF4α FACS analyses in undifferentiated human ESCs and HuH-7 cell line.** Undifferentiated ESCs and the

hepatocarcinoma cell line HuH-7 were used as positive control for CRIPTO-1 and HNF4α respectively. Examples of flow cytometry analysis showing **(A)** 71.10% of the undifferentiated ESCs expressing CRIPTO-1 and **(B)** 88.00% of the HuH-7 cell line positive for HNF4α.

Supplementary Figure 4: NKX2.5 **and *cMyHC* gene expression of human ESC-CMs.** Inhibition of the Wnt signaling pathway by IWR-1 was necessary for CM differentiation. **(A)** Bright field and fluorescent images of EBs at day 8 of cardiac differentiation. The NKX2.5$^{eGFP/w}$ human ESC line was used as a reporter cell line to visualize the NKX2.5 protein expression during differentiation. **(B)** *NKX2.5* gene expression at day 7 and 10 of differentiation. **(C)** *cMyHC* gene expression at day 10 and 18 of cardiac differentiation. Each data point is represented as ΔCt, normalized for the housekeeping genes *GAPDH, HPRT* and *RPL13a*. Data are representative of three independent experiments and values are expressed as mean ± SEM. Significant differences are indicated as $P < 0.001$: *** vs. control. Scale bar = 100 µm.
