# Supplementary material for: Activin A Modulates CRIPTO-1/HNF4α+ Cells to Guide Cardiac Differentiation from Human Embryonic Stem Cells
Source: Stem Cells Int. 2017 Jan 9;2017:4651238. doi: 10.1155/2017/4651238 (PMC5253508; doi:10.1155/2017/4651238)
Supplement: Supplementary file 1 — Supplementary Video 1: Embryoid body (EB) formation and morphology of human embryonic stem cells (ESCs) during in vitro cardiac differentiation. High doses of ActA (50 and 100 ng/mL ActA) increased beating frequency and contracting area of EBs. Representation of EBs at day 10 of cardiac differentiation of human ESCs (A) under control conditions (without ActA), with (B) 10 ng /mL ActA, (C) 25 ng/mL ActA, (D) 50 ng/mL ActA, and (E) 100 ng/mL ActA. Contracting areas are indicated by white dashed lines. [file 4651238.f1.zip › duelen et al. - supplementary table_sci_1816896.pdf]

Supporting Information Table

Supplementary Table S1: Primer sequences used in RT-qPCR analysis.

Gene	Primer	Primer sequences 5' > 3'
ACVR1B (ALK4)	Forward	CCCTCTTTGTCCAGCGCACAG
	Reverse	ATCAAACAGGGACCCGTGCTCAT
ACVR2A	Forward	ATCACAAGATGGCCTACCCTC
	Reverse	CCAGGCAAACTGTAGACTTCGTA
ACVR2B	Forward	TGGCTGTTCGGTTTGAG
	Reverse	CGTCTCTCTGGAAGTTGATG
BRACH	Forward	ACCCAGTTCATAGCGGTGAC
	Reverse	AAGCTTTTGCAAATGGATTG
cMyHC	Forward	GCCCTTTGACATTCGCACTG
	Reverse	CGGGACAAAATCTTGGCTTTGA
CRIPTO-1 (TDGF1)	Forward	CCCTCCTTCTACGGACGGAA
	Reverse	CAGGGAACACTTCTTGGGCAG
GAPDH	Forward	TCAAGAAGGTGGTGAAGCAGG
	Reverse	ACCAGGAAATGAGCTTGACAAA
HNF4α	Forward	ACTACGGTGCCTCGAGCTGT
	Reverse	GGCACTGGTTCCTCTTGTCT
HPRT	Forward	TGACACTGGCAAAACAATGCA
	Reverse	GGTCCTTTTCACCAGCAAGCT
INHBA (ActA)	Forward	ACGGGTATGTGGAGATAGAGGA
	Reverse	GGACTTTTAGGAAGAGCCAGACT
NANOG	Forward	TGGCCGAAGAATAGCAATGGTGTG
	Reverse	TTCCAGGTCTGGTTGCTCCACATT
NKX2.5	Forward	ACCTCAACAGCTCCCTGACTCT
	Reverse	ATAATCGCCGCCACAAACTCTCC
NODAL	Forward	CAGTACAACGCCTATCGCTGT
	Reverse	TGCATGGTTGGTCGGATGAAA

OCT4	Forward	CGAGCAATTTGCCAAGCTCCTGAA
	Reverse	GCCGCAGCTTACACATGTTCTTGA
PAX6	Forward	AACGATAACATACCAAGCGTGT
	Reverse	GGTCTGCCCGTTCAACATC
RPL13a	Forward	CCTGGAGGAGAAGAGGAAAGAGA
	Reverse	TTGAGGACCTCTGTGTATTTGTCAA
SOX1	Forward	GCAAGATGGCCCAGGAGAA
	Reverse	CCTCGGACATGACCTTCCA
SOX17	Forward	GTGGACCGCACGGAATTTG
	Reverse	GGAGATTCACACCGGAGTCA
TNNT2	Forward	ACAGAGCGGAAAAGTGGGAAG
	Reverse	TCGTTGATCCTGTTTCGGAGA
