# Supplementary material for: Activin A Modulates CRIPTO-1/HNF4α+ Cells to Guide Cardiac Differentiation from Human Embryonic Stem Cells
Source: Stem Cells Int. 2017 Jan 9;2017:4651238. doi: 10.1155/2017/4651238 (PMC5253508; doi:10.1155/2017/4651238)
Supplement: Supplementary file 1 — Supplementary Video 1: Embryoid body (EB) formation and morphology of human embryonic stem cells (ESCs) during in vitro cardiac differentiation. High doses of ActA (50 and 100 ng/mL ActA) increased beating frequency and contracting area of EBs. Representation of EBs at day 10 of cardiac differentiation of human ESCs (A) under control conditions (without ActA), with (B) 10 ng /mL ActA, (C) 25 ng/mL ActA, (D) 50 ng/mL ActA, and (E) 100 ng/mL ActA. Contracting areas are indicated by white dashed lines. [file 4651238.f1.zip › duelen et al. - supplementary video legends_sci_1816898.pdf]

Supporting Information Video Legends

Supplementary Video 1: **Embryoid body (EB) formation and morphology of human embryonic stem cells (ESCs) during *in vitro* cardiac differentiation.** High doses of ActA (50 and 100 ng/mL ActA) increased beating frequency and contracting area of EBs. Representation of EBs at day 10 of cardiac differentiation of human ESCs **(A)** under control conditions (without ActA), with **(B)** 10 ng /mL ActA, **(C)** 25 ng/mL ActA, **(D)** 50 ng/mL ActA and **(E)** 100 ng/mL ActA. Contracting areas are indicated by white dashed lines.

Supplementary Video 2: **Human ESC-derived CMs (ESC-CMs) differentiation potential after CRIPTO-1 blockage.** CRIPTO-1 blocking peptide (BP) impaired EB formation in ActA-directed *in vitro* cardiac differentiation. ActA (100 ng/mL) pretreated EBs at day 13 of differentiation **(A)** in the control condition and **(B)** after CRIPTO-1 BP treatment. Contracting areas are indicated by white dashed lines.

Supplementary Video 3: **Intracellular Ca^{2+} handling in human ESC-CMs.** Ca^{2+} dynamics of ESC-CMs was assessed at day 40 using a Ca^{2+}-sensitive fluorescent indicator Fluo-4. **(A)** Ca^{2+} handling in differentiated CMs under control conditions (without ActA).
