# Supplementary figures and images for: Activin A Modulates CRIPTO-1/HNF4α+ Cells to Guide Cardiac Differentiation from Human Embryonic Stem Cells
Source: Stem Cells Int. 2017 Jan 9;2017:4651238. doi: 10.1155/2017/4651238 (PMC5253508; doi:10.1155/2017/4651238)

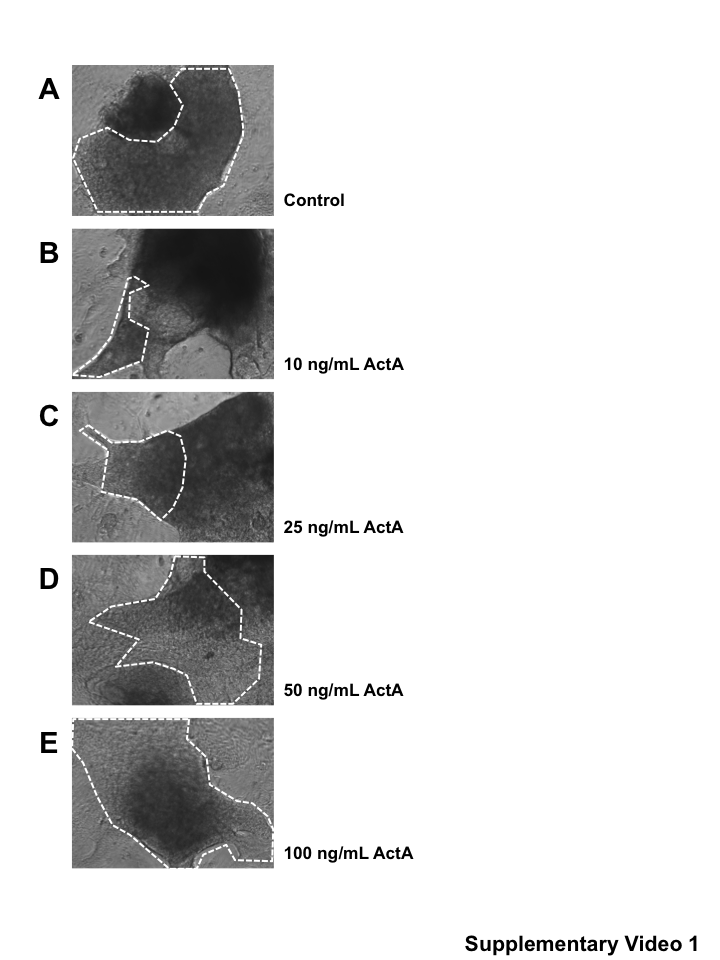

Supplement: Supplementary file 1 — Supplementary Video 1: Embryoid body (EB) formation and morphology of human embryonic stem cells (ESCs) during in vitro cardiac differentiation. High doses of ActA (50 and 100 ng/mL ActA) increased beating frequency and contracting area of EBs. Representation of EBs at day 10 of cardiac differentiation of human ESCs (A) under control conditions (without ActA), with (B) 10 ng /mL ActA, (C) 25 ng/mL ActA, (D) 50 ng/mL ActA, and (E) 100 ng/mL ActA. Contracting areas are indicated by white dashed lines. [file 4651238.f1.zip › suppvideo 1a-e (snapshot)_sci_1816941.tiff]

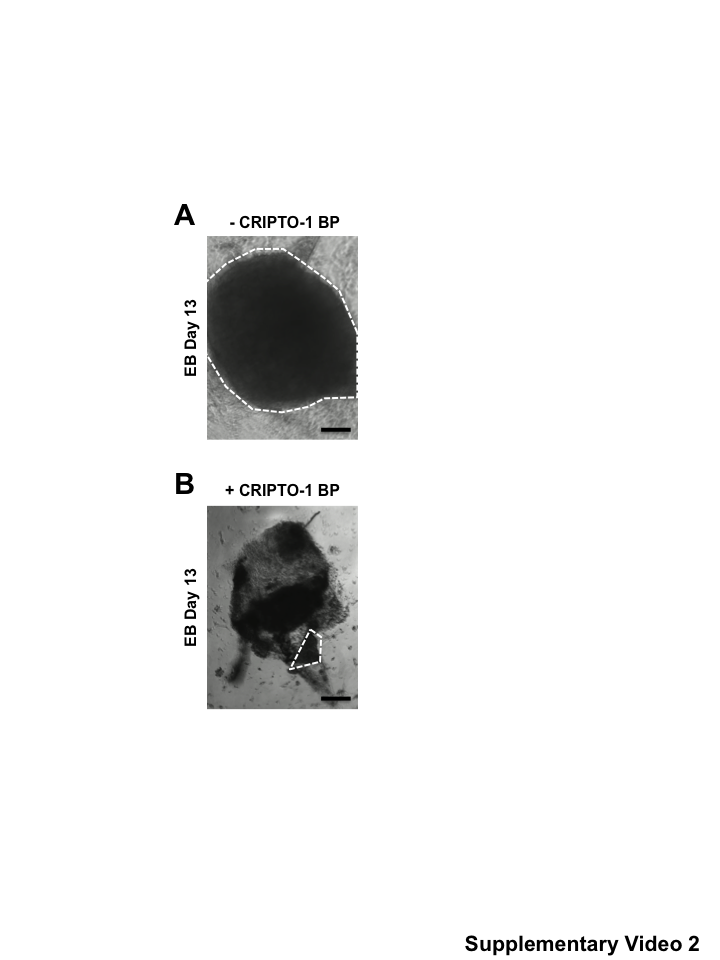

Supplement: Supplementary file 1 — Supplementary Video 1: Embryoid body (EB) formation and morphology of human embryonic stem cells (ESCs) during in vitro cardiac differentiation. High doses of ActA (50 and 100 ng/mL ActA) increased beating frequency and contracting area of EBs. Representation of EBs at day 10 of cardiac differentiation of human ESCs (A) under control conditions (without ActA), with (B) 10 ng /mL ActA, (C) 25 ng/mL ActA, (D) 50 ng/mL ActA, and (E) 100 ng/mL ActA. Contracting areas are indicated by white dashed lines. [file 4651238.f1.zip › suppvideo 2a-b (snapshot)_sci_1816944.tiff]

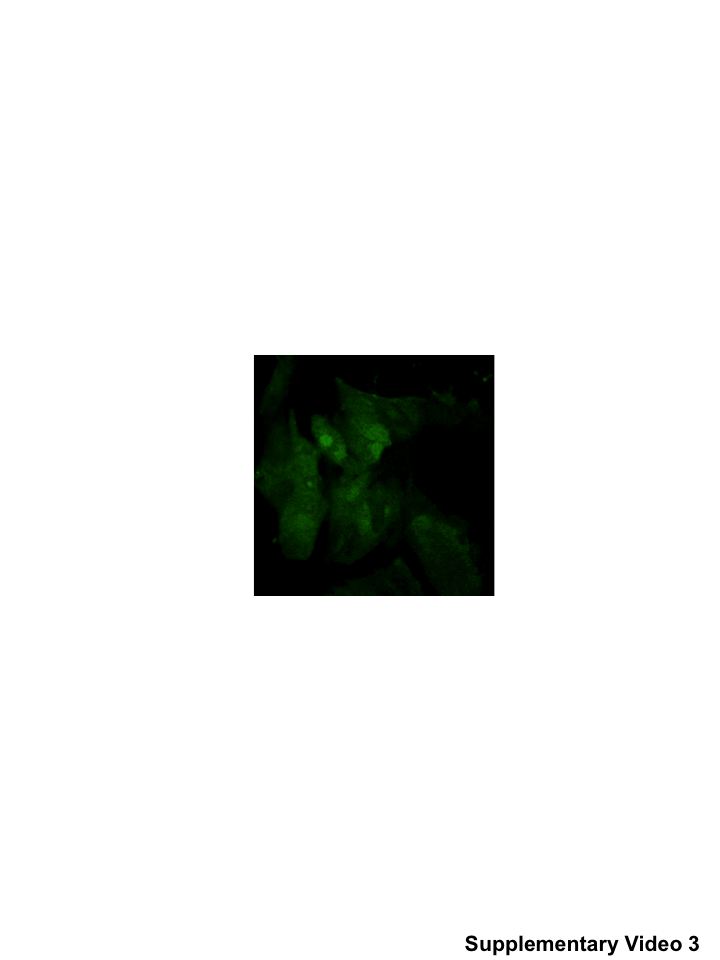

Supplement: Supplementary file 1 — Supplementary Video 1: Embryoid body (EB) formation and morphology of human embryonic stem cells (ESCs) during in vitro cardiac differentiation. High doses of ActA (50 and 100 ng/mL ActA) increased beating frequency and contracting area of EBs. Representation of EBs at day 10 of cardiac differentiation of human ESCs (A) under control conditions (without ActA), with (B) 10 ng /mL ActA, (C) 25 ng/mL ActA, (D) 50 ng/mL ActA, and (E) 100 ng/mL ActA. Contracting areas are indicated by white dashed lines. [file 4651238.f1.zip › suppvideo 3 (snapshot)_sci_1816946.tiff]
